# Sepsis in multimorbidity: a domain-based framework for systemic vulnerability and precision care

**DOI:** 10.3389/fmed.2026.1827818

**Published:** 2026-04-28

**Authors:** Jhan S. Saavedra-Torres, Humberto Alejandro Nati-Castillo, Alice Gaibor-Pazmiño, Wilder Fernando Ortiz Erazo, María Alejandra Martínez Castaño, Cristhian Camilo Nieto Brandon, Diana Catalina Parra Ramos, Juan Villamil, Leonardo Sánchez S., Andrés López-Cortés, Juan S. Izquierdo-Condoy

**Affiliations:** 1 Grupo de Investigación en Educación y Salud (GINEYSA), Facultad de Salud, Universidad Santiago de Cali, Cali, Colombia; 2 One Health Research Group, Universidad de Las Américas, Quito, Ecuador; 3 Interinstitutional Group on Internal Medicine (GIMI 1), Department of Internal Medicine, Universidad Libre, Cali, Colombia; 4 Facultad de Ciencias de la Salud, Universidad del Quindío, Armenia, Colombia; 5 Facultad de Ciencias de la Salud, Corporacion Universitaria Alexander von Humboldt, Armenia, Colombia; 6 Cancer Research Group, Universidad de las Américas, Quito, Ecuador

**Keywords:** endothelial dysfunction, immunoparalysis, immunothrombosis, multimorbidity, sepsis

## Abstract

Sepsis is a leading cause of morbidity and mortality, amplified by multimorbidity. This narrative review synthesizes epidemiological, pathophysiological, and immunological evidence to show how prevalent conditions—type 2 diabetes and obesity, heart failure and cerebrovascular disease, COPD, chronic kidney disease, cancer/HIV, and severe mental illness—reshape sepsis biology and outcomes. Convergent mechanisms include low-grade inflammation, impaired innate and adaptive immunity, endothelial injury with immunothrombosis/NETosis, barrier disruption with dysbiosis, and neuroendocrine maladaptation. These processes drive an early hyperinflammatory peak followed by immunoparalysis, increasing risks of secondary infection, multiorgan dysfunction, and death. Malnutrition modulates trajectories, and nosocomial sepsis contributes disproportionately to mortality. We propose an integrative framework in which comorbidities differentially load risk across five domains—immunity/inflammation, endothelium, barriers/microbiota, neuroaxis, and immunometabolism—clarifying bedside heterogeneity and therapeutic tolerance. Clinical implications include mechanism- and phenotype-aligned care: titrated fluids and vasoactives for limited cardiac or renal reserve; PK/PD optimization and timely antimicrobial de-escalation in obesity and chronic kidney disease; and immune/organ monitoring (e.g., monocyte HLA-DR, NGAL/KIM-1). System priorities include stronger prevention bundles for hospital-acquired sepsis and post-sepsis follow-up. Research needs include endotyping and trials testing mechanism-matched therapies, alongside PK/PD studies and cohorts tracking neurocognitive and cardiometabolic outcomes. Viewing sepsis through multimorbidity enables personalized care and reduced long-term burden.

## 1 Introduction

Sepsis is a life-threatening condition characterized by a dysregulated inflammatory response to infection, which can progress to multiple organ dysfunction and death (1, 2). Its clinical and social relevance has increased in recent decades due to the growing prevalence of chronic comorbidities worldwide, such as diabetes mellitus, heart failure, chronic obstructive pulmonary disease (COPD), and chronic kidney disease (1, 3, 4). These conditions not only predispose individuals to a higher risk of developing sepsis but also complicate its clinical course and increase associated mortality (5–7).

The infections most frequently leading to sepsis are pneumonia and urinary tract infections. Sociodemographic factors such as advanced age, White race, low educational attainment, limited income, and smoking are also associated with increased incidence of sepsis (1, 2).

The underlying pathophysiology can be linked to persistent systemic inflammation and immune dysfunction observed in patients with chronic diseases, which compromise the host’s ability to mount an effective response against infections. Additionally, endothelial dysfunction in vascular diseases facilitates progression toward organ failure, a hallmark of sepsis (1, 2).

Chronic diseases substantially increase the risk of sepsis. Among the most strongly associated are chronic lung disease, peripheral artery disease, chronic kidney disease, prior myocardial infarction (1, 8), diabetes mellitus, stroke, deep vein thrombosis, coronary artery disease, hypertension, atrial fibrillation, and dyslipidemia (1, 3, 4). The risk rises proportionally with the number of comorbidities present (1).

These findings underscore the need for interventions aimed at rigorous control of chronic comorbidities and preventive strategies against infections as essential components to reduce the clinical and systemic burden of sepsis (1, 3, 4).

This review seeks to analyze the complex interplay between sepsis and chronic diseases, integrating epidemiological, pathophysiological, and immunological perspectives to provide an updated map of systemic vulnerability. The overarching goal is to elucidate how comorbidities modulate immune responses and clinical trajectories in sepsis, thereby identifying factors that may guide personalized therapeutic interventions.

Sepsis is one of the leading causes of morbidity and mortality worldwide, and its impact is exacerbated by multimorbidity. Metabolic comorbidities such as type 2 diabetes mellitus and obesity; cardiovascular conditions such as heart failure; pulmonary disorders such as COPD; renal diseases such as chronic kidney disease; as well as cancer, HIV, and severe mental illness, substantially influence the incidence, severity, and outcomes of sepsis. These comorbidities condition higher risks of hospitalization, complications, and mortality (9, 10). Nosocomial sepsis adds an additional burden, with high incidence in intensive care units (ICUs) and neonatal units and mortality rates exceeding 50% among critically ill patients (11–13). In parallel, survivors often experience persistent neurological and psychosocial sequelae, highlighting that sepsis is not merely an acute event but also a determinant of long-term vulnerability. This epidemiological landscape demonstrates that multimorbidity amplifies both the risk and the global burden of sepsis and emphasizes the urgent need for integrated strategies of prevention, surveillance, and management tailored to heterogeneous risk profiles (14, 15).

## 2 Immunological and pathophysiological bases of sepsis in the context of comorbidity

### 2.1 Dysfunctional immune response in sepsis

Sepsis begins when invading microorganisms such as bacteria, viruses, or fungi release pathogen-associated molecular patterns (PAMPs), which are recognized by innate immune receptors, particularly Toll-like receptors (TLRs) expressed on monocytes, macrophages, and dendritic cells. In parallel, tissue injury releases endogenous danger signals (damage-associated molecular patterns, DAMPs), which activate the same signaling pathways (6, 16, 17). This stimulation promotes the translocation of NF-κB into the nucleus, triggering the transcription of proinflammatory genes (5–7, 18).

As a result, there is a massive release of proinflammatory cytokines such as IL-1β, TNF-*α*, and IL-6, together with chemokines that recruit neutrophils to the site of infection (5, 17). Under physiological conditions, this response is self-limiting and promotes resolution. In sepsis, however, it becomes dysregulated and persistent, driving systemic inflammation, endothelial dysfunction, and increased capillary permeability, which clinically manifest as hypotension, edema, and multiorgan failure (5, 7, 16).

Neutrophils, as the first effector cells, release neutrophil extracellular traps (NETs), composed of DNA and antimicrobial proteins, that serve to immobilize pathogens. However, excessive NET formation activates coagulation and damages the endothelium, promoting microthrombosis, hypoperfusion, and progressive organ dysfunction (5, 7, 16).

At the same time, there is accelerated depletion of T and B lymphocytes, as well as dendritic cells, through apoptosis. This immunosuppressive phenomenon reduces host defense capacity, favoring secondary infections, viral reactivation, and prolongation of critical illness (5, 7, 16, 19).

Neurologically, microglial activation and neuroinflammation compromise the integrity of the blood–brain barrier, leading to septic encephalopathy. Clinically, this manifests as delirium, altered consciousness, and, in severe cases, coma (20–22).

**Figure 1:**
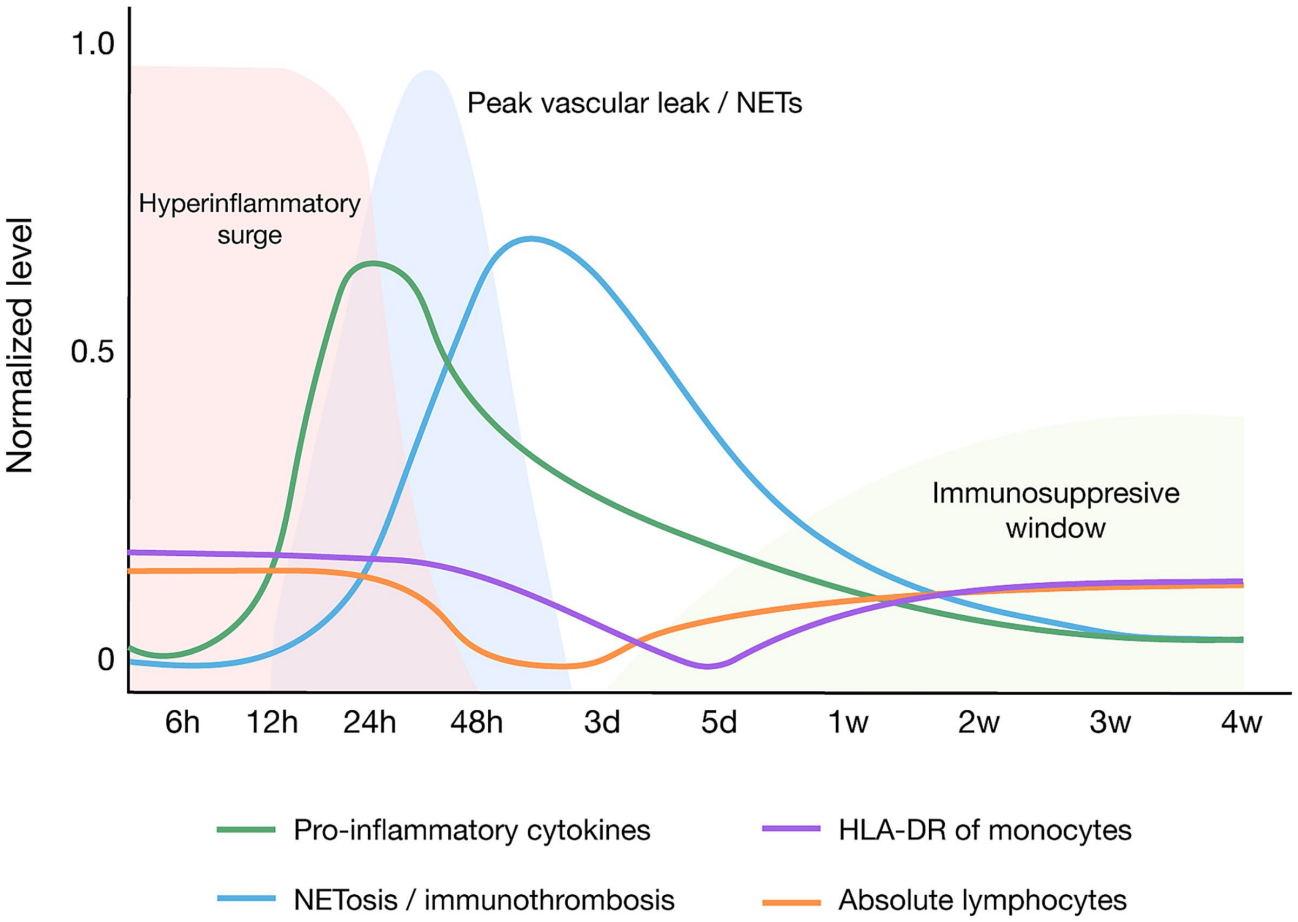
Baseline immunodynamic trajectory in sepsis. Schematic timing of key immune and hemostatic signals from symptom onset (day 0) through week 2: (i) Pro-inflammatory cytokines (e.g., IL-6, TNF-*α*, IL-1*β*), (ii) NETosis/immunothrombosis, (iii) monocyte HLA-DR expression, and (iv) absolute lymphocyte count. The early hyperinflammatory peak is followed by a variable immunosuppressive window marked by low HLA-DR and lymphopenia. Shaded region denotes typical ICU admission window. NETs, neutrophil extracellular traps.

The baseline timing of cytokine surge, peak NETosis/leak, and the subsequent immunosuppressive window is summarized in Figure 1.

### 2.2 Malnutrition, immunoparalysis, and post-sepsis musculoskeletal vulnerability

To orient disease-specific discussions, Table 1 consolidates how prevalent chronic comorbidities map onto five pathobiological domains—immunity/inflammation, endothelium, barriers/microbiota, neuroaxis, and immunometabolism—and highlights exemplar biomarkers with bedside implications.

**Table 1:** Activation of pathobiological domains across major chronic comorbidities—mechanisms, exemplar biomarkers, and bedside implications.

Comorbidity	Immune/Inflammation	Endothelium	Barriers and microbiota		Key biomarkers (examples)	Clinical implications
T2DM	IL-6/TNF-α ↑; NF-κB/JNK signaling; impaired neutrophil/T-cell responses	Endothelial dysfunction; microthrombosis	Dysbiosis; skin/urinary infections	—	HLA-DR ↓ (late); IL-6 ↑	Higher risk of sepsis and AKI; course modulated by glycemic control
Obesity	Leptin ↑, adiponectin ↕; RAAS activation; attenuated early cytokine surge	Fragile glycocalyx; prothrombotic state	Microbiota dysbiosis	—	Baseline CRP ↑	“Obesity paradox” (mortality ↓ with longer length-of-stay); thrombotic complications ↑
COPD	Dysfunctional neutrophils/macrophages; impaired innate defense	Leaky pulmonary capillary bed	Chronic colonization; biofilm	—	IL-6 ↑; neutrophilia	ARDS risk ↑; greater need for mechanical ventilation; recurrent severe pneumonia
CKD	Innate/adaptive immunity impaired	Venous congestion; flow–function dissociation	Bacterial translocation ↑	—	NGAL/KIM-1 ↑; HLA-DR ↓	Persistent AKI risk; reduced antimicrobial efficacy
HFrEF	Systemic inflammation; immune-checkpoint downregulation	Permeability ↑; tissue edema	—	—	BNP/NT-proBNP ↑	High susceptibility to volume overload and congestion with standard fluids
Cancer/HIV	Neutropenia or IL-10–skewed response; CRP/PCT often low	Infectious endotheliitis	Fragile mucosae	—	NLR ↑; IL-10 ↑	Opportunistic pathogens; atypical presentations with diagnostic delay

Domains include: Immunity/Inflammation; Endothelium; Barriers/Microbiota; Neuroaxis; Immunometabolism. Arrows: ↑ increase; ↓ decrease; ↕ mixed/variable. T2DM, type 2 diabetes mellitus; COPD, chronic obstructive pulmonary disease; CKD, chronic kidney disease; HFrEF, heart failure with reduced ejection fraction; RAAS, renin–angiotensin–aldosterone system; CRP, C-reactive protein; ARDS, acute respiratory distress syndrome; AKI, acute kidney injury; NGAL, neutrophil gelatinase-associated lipocalin; KIM-1, kidney injury molecule-1; BNP/NT-proBNP, B-type natriuretic peptide/N-terminal pro-BNP; NLR, neutrophil-to-lymphocyte ratio; PCT, procalcitonin; HLA-DR, human leukocyte antigen–DR.

Nutritional status has been shown to play a pivotal role in sepsis outcomes. In septic patients admitted to ICUs, the prevalence of malnutrition has been reported as 42.8% when assessed by height indices and 33.3% when assessed by body weight (23). In ICU-based sepsis studies, 100% of patients evaluated exhibited hypoalbuminemia within the first days of hospitalization, while 93.3% demonstrated zinc deficiency. Additionally, 35% showed severe lymphocytopenia (<500 lymphocytes/mm^3^) on the first day of admission, a finding consistent with functional immunoparalysis; during clinical evolution, increases in serum zinc levels were accompanied by progressive recovery of lymphocyte counts (23–25). Similarly, among adults with sepsis, a higher 28-day survival rate was observed in patients who achieved caloric intake above 89% of daily requirements by day 7, and higher levels of HLA-DR expression on monocytes at day 1 were associated with better clinical prognosis. However, malnutrition did not alter mortality in patients with established immunosuppression, defined by low HLA-DR expression (25).

These findings can be better understood in light of the profound metabolic response triggered by sepsis. The hypermetabolic and catabolic stress response accelerates nutritional deterioration early in the disease course, thereby aggravating immune dysfunction, organ failure, and overall clinical complexity (26, 27). Sustained activation of the neuroendocrine stress axis and inflammatory signaling increases energy expenditure, insulin resistance, and preferential protein catabolism, while mitochondrial dysfunction and impaired cellular bioenergetics limit efficient substrate utilization and contribute to organ dysfunction (27, 28). In this context, nutritional deterioration is not merely a consequence of reduced intake, but also a manifestation of sepsis-induced immunometabolic reprogramming and anabolic resistance, with activation of proteolytic pathways that promote progressive muscle wasting and negative nitrogen balance (28, 29).

Objective nutritional risk has also emerged as an adverse prognostic signal in sepsis. In a large multicenter cohort, moderate and severe malnutrition defined by objective nutritional indices were associated with higher 30-day mortality, and this signal remained evident even among mechanically ventilated patients (30). However, the benefit of nutritional support is unlikely to depend on calorie delivery alone and should instead be understood within individualized strategies aligned with metabolic phase, illness severity, gastrointestinal tolerance, and recovery potential (31, 32). Thus, malnutrition may influence not only acute trajectories but also the pace of physical recovery and long-term vulnerability among survivors (31, 33, 34).

Beyond the acute phase, malnutrition converges with sarcopenia and broader musculoskeletal decline as part of a syndrome of post-sepsis vulnerability. A meta-analysis found that sarcopenia at admission was associated with increased early mortality in sepsis, and this adverse signal persisted after discharge at 3–6 months and at 1 year (35). Likewise, pre-existing low muscle mass independently predicted worse 1-year outcomes in critically ill patients with sepsis, suggesting that muscle depletion may be more strongly linked to long-term vulnerability than to in-hospital survival alone (36). An emerging osteometabolic dimension may further shape survivorship trajectories: through an osteoimmunologic framework, systemic inflammation, immobilization, endocrine dysregulation, and altered calcium–vitamin D homeostasis may shift bone remodeling toward resorption while impairing osteoblast activity and skeletal repair (37–39). Observational data suggests a bidirectional relationship in which lower bone mineral density is associated with increased future risk of infection and sepsis, whereas sepsis itself may increase subsequent risk of osteoporosis and fracture (40–42). Although this area remains less developed than the evidence on sarcopenia, it reinforces the concept that malnutrition, muscle loss, and bone fragility converge within a common network of systemic vulnerability and deserve greater consideration in longitudinal prognostic assessment and structured post-sepsis follow-up (43, 44).

## 3 Metabolic and endocrine comorbidities

### 3.1 Diabetes mellitus

Type 2 diabetes mellitus (T2DM) is associated with a significantly higher risk of infection and sepsis, with studies reporting a 2- to 6-fold increased risk compared to non-diabetic individuals (14, 15), along with elevated morbidity and mortality rates (45–47). Increased colonization with resistant pathogens, particularly methicillin-resistant *Staphylococcus aureus* (MRSA), has been documented. Over a 25-year period, diabetes was present in 17% of hospitalized patients with sepsis and represented the most common comorbidity among those who developed complications and died after a septic episode (9, 10, 47).

From a pathophysiological perspective, T2DM creates an altered immunometabolic environment that promotes sepsis progression. Chronic low-grade inflammation, mediated by IL-6, TNF-*α*, and IL-1β, activates pathways such as NF-κB and JNK, driving endothelial dysfunction, insulin resistance, and leukocyte trafficking alterations (48–50). This state translates into an increased risk of acute kidney injury (AKI), secondary to hypoperfusion, endothelial inflammation, and preexisting susceptibility, while the association with acute respiratory distress syndrome (ARDS) remains controversial (51, 52).

T2DM also induces specific immunological alterations, including neutrophil dysfunction, humoral immune defects, and abnormal T-cell responses, which reduce antimicrobial efficacy and facilitate severe infections (53–55). Although colonization with resistant pathogens, particularly MRSA, is more frequent in this group, this finding is considered secondary compared with the overall impact of poor glycemic control (10, 45–47). Interestingly, recent studies suggest that chronic exposure to hyperglycemia may generate immune adaptations that, in certain contexts, are associated with lower mortality among septic diabetic patients. However, this observation is preliminary and requires further validation (56, 57).

Therapeutically, hypoglycemic agents such as insulin, metformin, and thiazolidinediones have shown, in some studies, a potential to reduce the incidence and mortality of sepsis. Nevertheless, current evidence remains insufficient to establish definitive recommendations regarding their specific use or the optimal glycemic control targets during the septic course (48, 58).

### 3.2 Obesity and the obesity paradox

Obesity and its relationship with sepsis represent an area of growing clinical and pathophysiological interest. This highly prevalent condition among critically ill patients has been consistently associated with an increased risk of severe infections and sepsis development (59–63). The association is not explained solely by adipose tissue mass but also by the immunometabolic dysfunction inherent to obesity and the frequent coexistence of comorbidities such as T2DM, hypertension, cardiovascular disease, and renal failure. However, its impact on sepsis mortality has given rise to a controversial phenomenon known as the “obesity paradox” (59, 60).

This paradox refers to the observation that obese patients with sepsis exhibit lower short- and long-term mortality compared with those of normal or low body weight. Although associated with improved survival, this does not necessarily translate into early functional recovery: obese patients often experience longer hospitalizations and, in some cases, prolonged mechanical ventilation or ICU stays, which may reflect extended survival at the cost of higher complication burdens (59, 61, 62).

From a pathophysiological standpoint, several mechanisms have been proposed to explain this differential response. First, the greater energy reserves provided by adipose tissue may offer an adaptive advantage during the hypercatabolic phase of sepsis by sustaining cellular metabolism, nutritional stability, and partial preservation of immune function under prolonged stress. Additionally, obese patients display an attenuated inflammatory response during early sepsis, with lower levels of cytokines such as IL-6 and TNF-α, potentially mitigating the damage induced by the initial inflammatory storm (60, 63). Adipose tissue also acts as an immunomodulatory organ, capable of sequestering lipopolysaccharide (LPS) and other bacterial products, thereby buffering their systemic impact (64, 65).

Hormonally, adipocytes in obese individuals secrete leptin and adiponectin at elevated concentrations. Leptin plays a central role in immune modulation, activating T lymphocytes (particularly CD4^+^ T cells) and macrophages, and promoting Th1/Th17-driven inflammatory responses, whereas adiponectin exerts anti-inflammatory effects, attenuating macrophage activation and regulating metabolic homeostasis through pathways involving SIRT1 and AMPK (63, 66). Another relevant factor is the hyperactivity of the renin–angiotensin–aldosterone system (RAAS), common in obesity. While RAAS contributes to hypertension, its activation may provide hemodynamic advantages during sepsis, such as more effective responses to endogenous vasoconstrictors and reduced vasopressor requirements, facilitating circulatory stability in critical phases (59, 61, 62).

Nonetheless, this supposed adaptive advantage does not eliminate the clinical risks associated with obesity. Obesity increases susceptibility to more severe and difficult-to-manage infections—particularly of the skin, soft tissues, and surgical sites—due to impaired barrier function, chronic inflammation, and compromised lymphatic drainage. Furthermore, obesity induces significant alterations in the pharmacokinetics and pharmacodynamics of antimicrobials, including increased volume of distribution, altered hepatic metabolism, and modified renal clearance. These changes may reduce therapeutic efficacy and increase the risk of suboptimal exposure or cumulative toxicity (67–69).

The coexistence of cardiovascular, renal, and metabolic comorbidities, combined with insulin resistance, dyslipidemia, and persistent systemic inflammation, heightens the risk of multiple organ dysfunction and thromboembolic complications. This profile explains why obesity can simultaneously act as a source of adaptive reserve and a driver of organ dysfunction (59–63).

Therefore, the therapeutic approach to obese patients with sepsis should be individualized and multidisciplinary. It is essential to adjust antimicrobial and sedative dosing, closely monitor cardiovascular and respiratory function, and continuously evaluate nutritional and metabolic status. Early rehabilitation strategies and secondary prevention after recovery from the septic episode should also be prioritized (59–63).

Building on Figure 1 and Table 2 summarizes how common comorbidities deform key immune and hemostatic trajectories in sepsis (cytokines, NETosis, monocyte HLA-DR, and absolute lymphocytes), linking curves to practical bedside notes.

**Table 2:** Effects of chronic comorbidities on key immune and hemostatic curves in sepsis (Cytokines, NETosis, Monocyte HLA-DR, Lymphocytes).

Comorbidity	Pro-inflammatory cytokines	NETosis/Immunothrombosis	Monocyte HLA-DR	Absolute lymphocytes	Key clinical note
T2DM/Obesity	↑ higher early peak with slower decline (0–48 h)	Sustained ↑; prolonged tail to D7–D10	↓ moderate; slow recovery	↓ mild–moderate; delayed recovery if glycemic control is poor	Sustained capillary leak, persistent microthrombosis; “obesity paradox”: ↓ early mortality but ↑ complications/length of stay
COPD	↔, ↑ moderate	↑↑ higher and prolonged (24–72 h)	↓ mild–moderate (with recurrent infections)	↔, ↓ variable	ARDS risk, challenging ventilation; chronic colonization and macrophage dysfunction
CKD/HFrEF	Similar peak; prolonged endothelial inflammation if congestion	↑ with slower decline under volume overload	↓ with slow recovery	↓ mild; slow recovery (uremia, hemodilution)	Widened transition (48 h–D7); uncertain response to fluids (±); risk of edema and renal flow–function mismatch
Cancer/HIV	Atypical profile: IL-10↑ with low CRP/PCT (may mask severity)	Variable (depends on opportunistic pathogens)	↓↓ deeper nadir with very slow recovery	↓↓ more severe lymphopenia with delayed recovery	Extended immunosuppressive window (D3–Week 2–4); high risk of opportunistic infections/reactivations

D = day; W = week; LoS = length of stay; symbols as above. Link the rightmost column to specific bedside implications to guide clinicians in interpreting trajectories.

## 4 Cardiovascular comorbidities

### 4.1 Heart failure with reduced ejection fraction (HFrEF)

In patients with sepsis, the coexistence of HFrEF exacerbates cardiovascular dysfunction through converging immunometabolic mechanisms. Sepsis can induce or decompensate heart failure via mitochondrial dysfunction, oxidative stress, cardiomyocyte apoptosis, and sustained systemic inflammation. Recently, a specific gene signature—*GNMT, SEMA4B, FURIN, RNASE2, BEX1,* and *EPHX2*—with an area under the curve (AUC) greater than 0.9 has been identified, characterizing a shared immunopathological response between sepsis and HFrEF. This activation reflects structural injury, impaired myocardial contractility, and persistence of antigenic stimuli, compromising functional reserve under septic stress (70).

This immunometabolic dysfunction is also mirrored in the downregulation of immune checkpoints such as PD-1 and CTLA-4, observed in both murine models and human cardiac tissue. The loss of immunoregulatory mechanisms facilitates persistent antigenic activation, perpetuating systemic inflammation and directly contributing to myocardial damage. This dysfunctional environment not only compromises cardiac function during the acute septic phase but is also associated with worse long-term outcomes after hospitalization (70).

Consequently, the 90-day readmission rate after discharge reaches 30.2% in patients with HFrEF, compared with 22.5% in those without heart failure, representing a relative increase of 15% (71, 72). This vulnerability extends beyond the acute episode, negatively impacting prognosis, hospital stay, and health-care costs (71, 73, 74), thereby contributing to a substantial clinical and economic burden (75).

The underlying pathophysiology explains this adverse association: ventricular dysfunction limits the heart’s functional reserve, impairing its ability to adapt to the increased metabolic and fluid demands induced by sepsis (71, 73, 74). In this context, aggressive fluid administration—central to the initial management of septic shock—can precipitate volume overload, exacerbate congestion, and decompensate cardiac function in HFrEF patients (75–78).

Additional risk arises from the frequent requirement for vasopressors and inotropes to maintain tissue perfusion and blood pressure during septic shock. However, agents such as dopamine have been linked to higher risks of arrhythmias and adverse cardiovascular events, whereas norepinephrine has demonstrated a more favorable profile in terms of efficacy and safety (79–82). The presence of HFrEF further increases the likelihood of critical complications, including arrhythmias requiring cardioversion, pulmonary edema necessitating ventilatory support, and acute kidney injury requiring renal replacement therapy, either due to hypoperfusion or cardiorenal syndrome (79, 83, 84). In this setting, the chronic use of *β*-blockers must be carefully evaluated. Current evidence supports their continuation in the absence of acute hemodynamic instability, without associations with higher mortality or clinical deterioration (73).

Moreover, HFrEF is characterized by immune dysfunction that impairs the inflammatory response and hinders infection control, thereby promoting multiorgan deterioration (73, 75, 85). This convergence of cardiovascular and immune dysfunction defines a high-risk clinical phenotype in which sepsis acts as a trigger for systemic decompensation, marked by persistent hemodynamic instability, susceptibility to nosocomial infections, and poor organ recovery even after resolution of the acute episode (73).

### 4.2 Cerebrovascular disease and sepsis

Sepsis induces multifactorial neurological dysfunction characterized by ischemic and hemorrhagic cerebrovascular complications, which may occur during both the acute and delayed phases, substantially contributing to morbidity and mortality. A retrospective study analyzing 278 cranial CT scans in critically ill patients with suspected sepsis found that 10.8% presented significant acute cerebral findings. Among these, 7.2% corresponded to major cerebrovascular events, with a predominance of intracranial hemorrhages (80%) over ischemic strokes (20%). In addition, 4.3% showed diffuse parenchymal injury, mainly attributable to post–cardiac arrest hypoxia (86, 87).

The frequency of these findings was significantly higher in patients with deep coma (Glasgow ≤8), reaching 13.4%, compared with 4.4% in those with milder neurological compromise. Incidence also varied by clinical setting, being highest among patients from cardiac or nephrology ICUs (16.3%) compared with those from pulmonary (4.9%) or surgical units (6.1%) (86, 87).

Sepsis affects the central nervous system through complex pathophysiological mechanisms that extend beyond septic encephalopathy. Generalized endothelial dysfunction, inflammation-induced coagulopathy, cerebral vasoplegia, and impaired autoregulation of perfusion contribute to severe neurological events. At the microvascular level, capillary thrombosis, endothelial leakage, and glial cell activation exacerbate cerebral edema and perpetuate regional hypoperfusion. New-onset atrial fibrillation (AF) during sepsis, observed in up to 25% of critically ill patients, represents an adverse prognostic marker. Its occurrence reflects acute cardiovascular dysfunction, systemic inflammation, and dysregulated neurohumoral activation, particularly in patients with structural heart disease. This state amplifies thromboembolic and neurological risks, warranting careful consideration of the balance between anticoagulation and hemostatic safety, integrating factors such as hepatorenal function, inflammatory status, and hematologic profile (86–88).

In a retrospective study of patients with septic shock and AF, therapeutic anticoagulation was not associated with a significant reduction in clinically evident strokes or with an increase in major hemorrhages. However, it was linked to reduced in-hospital mortality, possibly due to prevention of subclinical embolic phenomena and modulation of disseminated intravascular coagulation (86, 87). Notably, this benefit coexisted with significant declines in hemoglobin levels among anticoagulated patients, necessitating interventions such as transfusions or therapeutic adjustments, reinforcing the need for close monitoring (89, 90).

Sepsis is increasingly recognized as a systemic trigger for cerebrovascular events, even in the post-acute period. In a cohort of over 120,000 septic patients, 0.5% experienced stroke within the following year, with associated risk factors including valvular heart disease, heart failure, chronic kidney disease, lymphoma, peripheral vascular disease, pulmonary disorders, and coagulopathy. Each additional point on a composite risk index was associated with a 43% increase in stroke probability, with the highest risk seen in younger patients without prior vascular history (89, 90).

This risk extends beyond the immediate post-sepsis period. A meta-analysis including more than 800,000 patients confirmed a robust association between sepsis and higher long-term stroke incidence, particularly among those with comorbidities such as diabetes mellitus, hypertension, and chronic kidney disease. These findings underscore the need for secondary prevention strategies, early neurological screening, and long-term surveillance in sepsis survivors (89, 90).

## 5 Pulmonary comorbidities

### 5.1 Chronic obstructive pulmonary disease (COPD)

Chronic obstructive pulmonary disease (COPD) is an independent risk factor that increases mortality, complications, and health-care resource utilization in patients with sepsis (12, 91, 92). Large cohort studies have shown that septic patients with COPD have higher 28-day mortality, greater need for invasive and noninvasive ventilation, higher SOFA and APACHE II scores, as well as increased ICU admissions and longer hospital stays (12, 91–93).

The link between COPD and sepsis is mediated by a complex interaction among chronic pulmonary inflammation, mucociliary dysfunction, and both local and systemic immune alterations. The innate immune response is impaired in these patients: alveolar macrophage phagocytosis is reduced, neutrophil activity is dysregulated, and antimicrobial peptide production is diminished. These changes facilitate chronic bacterial colonization and heightened susceptibility to invasive infections, particularly by *Haemophilus influenzae*, *Streptococcus pneumoniae*, and *Pseudomonas aeruginosa* (12, 92, 93).

Prolonged corticosteroid use, common in COPD exacerbations, adds an additional layer of immunosuppression and increases the risk of severe respiratory infections and sepsis. This vulnerability is compounded by the frequent coexistence of comorbidities such as heart failure, pulmonary hypertension, and diabetes mellitus, which worsen systemic outcomes (12, 92, 93).

Population-based studies have demonstrated that COPD patients who develop sepsis have significantly higher rates of hospitalization for pneumonia, more frequent recurrence of acute exacerbations, and greater medium-term mortality, even after hospital discharge, compared with COPD patients without sepsis (91, 93).

This pathological combination not only intensifies the acute infectious episode but also accelerates long-term respiratory and systemic decline (12, 92, 93). From a pathophysiological perspective, dysregulated inflammation in COPD is amplified during sepsis, with excessive release of proinflammatory cytokines, increased pulmonary capillary permeability, and elevated risk of acute respiratory distress syndrome (ARDS). This inflammatory storm prolongs organ dysfunction, hampers clinical recovery, and increases the risk of ventilatory failure, tracheostomy, and long-term dependence on oxygen or home ventilation (91, 93).

Furthermore, chronic hypoxia inherent to COPD can negatively modulate mitochondrial function and the immunometabolic response, thereby exacerbating sepsis-induced immunosuppression. In this setting, infection resolution may be impaired, leading to higher incidence of nosocomial infections and broader-spectrum antibiotic requirements (12, 92, 93).

In COPD patients, sepsis is strongly associated with an increased burden of respiratory and systemic sequelae. Survivors experience higher rates of severe exacerbations, hospitalizations, emergency visits, pneumonia, and severe pneumonia, as well as higher overall mortality. These findings indicate that sepsis represents a clinical inflection point, worsening the natural course of COPD and amplifying post-sepsis disease burden (91, 94).

## 6 Renal comorbidities

### 6.1 Chronic kidney disease (CKD)

Chronic kidney disease (CKD) is a frequent and highly relevant comorbidity in patients with sepsis, exerting an adverse impact on both short- and long-term outcomes. Sepsis-induced acute kidney injury (AKI) is a common complication, with incomplete recovery in nearly 47% of cases at hospital discharge or within 90 days, thereby accelerating progression to advanced CKD stages, increasing the need for renal replacement therapy (RRT), and elevating mortality (95–97).

Intermediate and advanced stages of AKI (stages 0C and ≥1) are associated with adjusted hazard ratios of up to 3.25 for major adverse renal events and death, underscoring the importance of stratified assessment of renal injury in the septic context (95, 97, 98).

From a pathophysiological perspective, the septic kidney is exposed to multiple insults: dysregulated systemic inflammation, endothelial dysfunction, heterogeneous hypoperfusion, altered renal microcirculation, and mitochondrial dysmetabolism (95, 99, 100). These mechanisms converge to produce acute tubular injury, cellular apoptosis, and loss of glomerular autoregulation (101–103), in a scenario characterized by “flow–function dissociation,” where renal blood flow may be preserved or even increased, but effective utilization of oxygen and nutrients remains impaired (95, 97, 98).

In patients with preexisting CKD, reduced renal functional reserve increases vulnerability to septic insult. This is further compounded by baseline proinflammatory status, acid–base disturbances, and mineral–bone abnormalities that impair immune responses and promote multiorgan dysfunction. These patients also display blunted febrile responses, greater bacterial dissemination, and reduced efficacy of antimicrobial therapy (95, 97, 98).

Initial resuscitation strategies in this population demand particular caution with fluid administration: volumes exceeding 27 mL/kg have been linked to longer hospital stays, higher need for mechanical ventilation, hemodynamic complications, and increased costs, without clear benefit in reducing vasopressor use or ICU admission (95, 97, 98). Fluid overload contributes to pulmonary edema, renal venous hypertension, and further glomerular deterioration (104).

Another challenge lies in scoring systems such as SOFA or qSOFA, which do not adequately adjust for baseline renal function or consider variability in prior glomerular filtration rate, leading to potential underestimation of severity and delays in critical interventions (105–107).

Accordingly, there is a pressing need to incorporate stratification models that integrate prior renal parameters, tubular injury biomarkers such as NGAL and KIM-1, and dynamic real-time renal monitoring tools (95, 99, 100). Their implementation would allow early identification of CKD patients at risk of progressive deterioration, optimization of nephroprotective measures, improved selection of timing and modality of RRT, and ultimately a reduction in sepsis-related morbidity and mortality (105–107).

## 7 Oncological and HIV comorbidities

### 7.1 Solid and hematologic malignancies

Sepsis is a frequent and critical complication in patients with malignancies and represents one of the leading causes of ICU admission. Up to 20% of critically ill patients have some form of cancer, with a tenfold higher risk of sepsis compared with the general population. Thirty-day mortality in this group remains high—around 40%—although it has declined in recent decades owing to advances in life support and clinical management strategies (108–110).

Hematologic malignancies such as acute leukemia, non-Hodgkin lymphoma, and multiple myeloma are the most common among septic oncology patients, with a significant proportion presenting with neutropenia at ICU admission. This condition, resulting from either the malignancy itself or chemotherapy, combined with functional defects in neutrophils, severely impairs host defense, hindering infection control and increasing the likelihood of multiple organ failure (108–110).

The most common infections are pneumonia, followed by abdominal infections, skin and soft tissue infections, and catheter-associated infections. Gram-negative bacilli predominate, with increasing antimicrobial resistance in nosocomial infections, further complicating management (108–110).

Prognostic factors influencing mortality include renal dysfunction, the severity of organ failure assessed by scores such as SOFA, the need for advanced life support (mechanical ventilation, continuous renal replacement therapy), and reduced adherence to standardized sepsis protocols. Biomarkers such as B-type natriuretic peptide and the neutrophil-to-lymphocyte ratio have demonstrated utility in outcome prediction (108–110).

Improving survival in this population requires not only effective infection control but also comprehensive care that accounts for tumor activity, functional status, and early definition of goals of care and appropriate levels of support (108–110).

### 7.2 HIV/AIDS

The introduction of combination antiretroviral therapy (cART) transformed HIV infection into a controllable chronic condition, yet sepsis remains a frequent and life-threatening complication with substantial morbidity and mortality (111, 112). The incidence of sepsis reaches up to 1,000 cases per 100,000 people with HIV, compared with 150–300 in the general population. This disparity persists even among patients with undetectable viral loads, due to residual immunosuppression, chronic opportunistic infections, and non-AIDS comorbidities such as COPD, liver disease, chronic kidney disease, and malignancies (112–114).

Hospital mortality among HIV-positive patients with sepsis is markedly higher, ranging from 55 to 58% at 6 months, compared with 27% in HIV-negative patients, reflecting both the clinical severity and the diagnostic and therapeutic challenges imposed by immune dysfunction (114, 115). Precipitating infections include not only common bacterial pathogens (Gram-negative bacilli) but also opportunistic organisms such as *Mycobacterium tuberculosis, Pneumocystis jirovecii, Cryptococcus neoformans,* and *Candida* spp., whose atypical presentations may delay diagnosis (108, 112–114).

From an immunological standpoint, HIV-positive patients exhibit an altered inflammatory response that directly influences sepsis progression. Classical biomarkers such as C-reactive protein (CRP) and procalcitonin (PCT) tend to be lower than in immunocompetent individuals, potentially masking infection severity. Conversely, persistently elevated interleukin-10 (IL-10)—an anti-inflammatory cytokine—suggests a shift toward a regulatory or tolerogenic immune response, characteristic of “immunoparalysis” observed in late sepsis and advanced immunodeficiency (112–114).

This immune imbalance translates into more aggressive clinical trajectories, with higher frequency of multiple organ dysfunction, prolonged vasopressor support, mechanical ventilation, and renal replacement therapy. Despite advances in critical care and the availability of cART, sepsis remains an independent predictor of both early and late mortality in HIV patients, even among those with partial immune recovery (112–114).

Clinical management of sepsis in HIV requires a multidisciplinary approach that integrates general sepsis protocols with immunocompromised-specific considerations. Early recognition, immediate initiation of broad-spectrum antimicrobials covering both common bacterial and opportunistic pathogens, and rational use of diagnostics for coinfections and reactivations are essential. Continuous evaluation of immune status—including viral load, CD4 + count, and latent coinfections—is critical to guiding therapy (112–114).

A particularly controversial issue is whether to initiate or reintroduce cART during the acute critical phase: while some studies suggest potential benefits from continuation, others warn of the risk of immune reconstitution inflammatory syndrome (IRIS). Currently, no definitive consensus exists (112–114).

## 8 Neuropsychiatric comorbidities

### 8.1 Severe mental illness (SMI)

Patients with severe mental illness (SMI)—including schizophrenia, bipolar disorder, and major depressive disorder—exhibit an unexpected clinical course in the setting of sepsis and septic shock. Although these conditions are generally associated with greater vulnerability and reduced access to health-care services, current evidence suggests lower mortality rates once hospitalized for sepsis compared with the general population without such disorders (116, 117).

A meta-analysis evaluating data from more than 2 million patients found substantially lower unadjusted hospital mortality among individuals with SMI, with an odds ratio (OR) of 0.61 (95% CI: 0.58–0.65), corresponding to nearly a 40% relative reduction in the risk of death during hospitalization for sepsis (116, 118).

A large multicenter study in France including nearly 188,000 patients diagnosed with septic shock reported lower 90-day mortality in those with SMI. Mortality was 32.2% in schizophrenia versus 45.5% in controls, 32.9% in bipolar disorder versus 45.3, and 41.4% in major depression versus 47.1%. These differences persisted after multivariable adjustment for age, sex, and comorbidities, with adjusted hazard ratios of 0.70 for schizophrenia and bipolar disorder and 0.85 for major depression (116–118).

In contrast, a Mendelian randomization study suggested that major depression may be modestly associated with increased risk of developing sepsis (OR 1.13; 95% CI: 1.02–1.26), pointing to heightened susceptibility to infection (116, 117). However, no significant associations were found between schizophrenia or bipolar disorder and sepsis onset, nor between any of these psychiatric conditions and sepsis-related mortality. These findings reinforce the notion that prior psychiatric history does not necessarily translate into higher risk of death once sepsis is established (116–118).

Several clinical and biological hypotheses have been proposed to explain this phenomenon. First, SMI patients admitted to ICUs are often younger and, in many cohorts, include a higher proportion of women—factors that may confer biological advantages. In addition, comorbidity profiles and treatment patterns differ significantly. Chronic use of antipsychotics, mood stabilizers, and certain antidepressants may modulate inflammatory and immune pathways. Some psychotropic medications are thought to exert immunomodulatory effects through dopamine, serotonin, and glucocorticoid receptors, thereby altering systemic inflammatory responses (116–118).

Moreover, atypical immune profiles in individuals with SMI—characterized by chronic glial activation, altered cytokine levels including IL-6, IL-10, and TNF-*α*, and changes in neuroendocrine signaling—may attenuate the hyperinflammatory response typical of severe sepsis. This could translate into reduced endothelial dysfunction, less cytokine-mediated tissue injury, and greater metabolic adaptability during septic shock, although these mechanisms remain speculative and require experimental validation (116–118).

Another contributing factor may be earlier recognition and treatment during acute episodes due to frequent contact with health-care services or assisted living facilities, which could enable more timely interventions against severe infections.

Taken together, these findings challenge the traditional paradigm associating SMI with poorer clinical outcomes. In the specific context of sepsis, patients with SMI appear to have a relative survival advantage, suggesting the presence of immunological or pharmacological protective mechanisms that remain incompletely understood (116–118).

## 9 Aggravating clinical contexts

### 9.1 Nosocomial infections and hospital-acquired sepsis

Hospital-acquired sepsis represents a substantial proportion of cases managed in inpatient settings. A meta-analysis of 51 studies estimated that 23.6% of patients with sepsis developed the infection during hospitalization. In ICUs, 24.4% of sepsis episodes with organ dysfunction originated in the hospital, and nearly half (48.7%) were of nosocomial origin (119–121).

Incidence rates are particularly high: 9.3 cases of hospital-acquired sepsis with organ dysfunction per 1,000 hospitalized patients have been reported, rising to 56.5 per 1,000 in ICUs. In neonatal intensive care units (NICUs), this figure exceeds 250 per 1,000 admissions. Mortality is equally alarming: in ICU-acquired sepsis with organ dysfunction, case fatality rates reach 52.3% (119–121).

These findings confirm that nosocomial sepsis constitutes a critical public health problem, exerting a disproportionate impact on critically ill patients and neonates. Variability across studies in both incidence and mortality highlights the urgent need to strengthen epidemiological surveillance, standardize clinical and microbiological definitions, and implement sustainable infection prevention and control strategies, with particular emphasis on ICUs and NICUs (119–121).

## 10 Discussion

**Figure 2:**
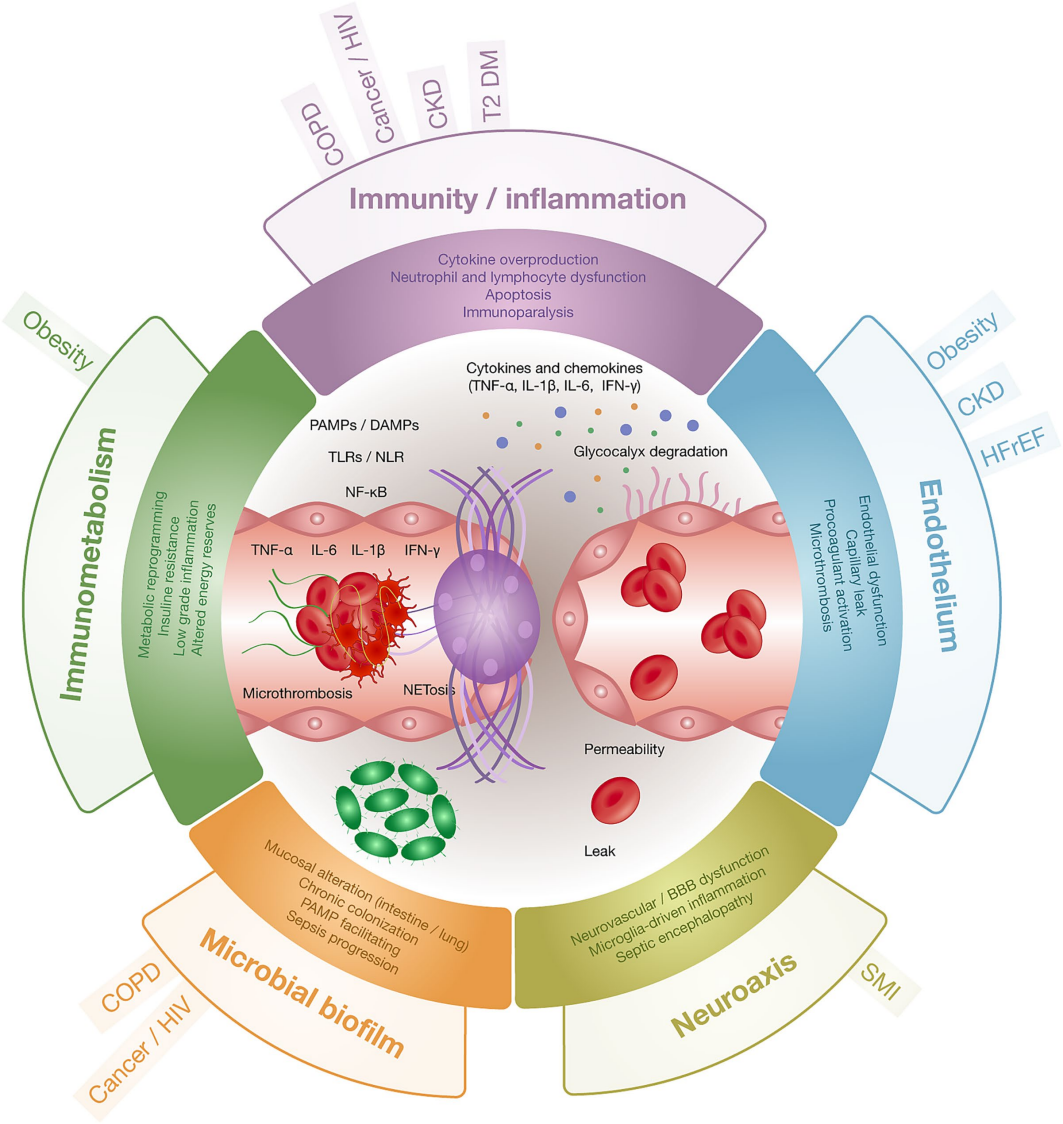
Integrative model of system-level vulnerability in sepsis: convergent domains and comorbidity modifiers. The innate-sensing axis (PAMPs/DAMPs → TLR/NLR → NF-κB) feeds five convergent domains—Immunity/Inflammation, Endothelium (glycocalyx), Barriers/Microbiota (e.g., gut, lung epithelium), Neuroaxis, and Immunometabolism. The microvascular-barrier unit is the final common pathway for cytokine-driven inflammation, NETosis/immunothrombosis, and vascular leak. Comorbidities differentially load risk across domains, shaping clinical trajectories. PAMPs, pathogen-associated molecular patterns; DAMPs, damage-associated molecular patterns; TLR, Toll-like receptor; NLR, NOD-like receptor; BBB, blood–brain barrier.

This review portrays sepsis amid chronic multimorbidity as a systems-level disorder arising from convergent axes of immune dysregulation, endothelial injury, barrier failure, and neuroendocrine maladaptation. The accumulation of chronic conditions—particularly type 2 diabetes mellitus, heart failure, COPD, chronic kidney disease, cerebrovascular disease, obesity, cancer, HIV, and severe mental illness—not only increases the likelihood of developing sepsis but also reshapes its clinical trajectory toward greater organ dysfunction and higher mortality. Mechanistically, a background of low-grade inflammation and impaired innate/adaptive immunity primes the host for an exaggerated early inflammatory surge with microvascular injury and immunothrombotic activity, followed by a phase of pronounced immunosuppression that sustains secondary infections and multi-organ failure. These convergent axes are synthesized in our proposed integrative model (Figure 2).

The marked heterogeneity observed at the bedside reflects shifting contributions of hyperinflammation and immunosuppression across time and across comorbidity profiles, in patterns that mirror the pathobiological domains summarized in Figure 1. These differences influence tolerance to resuscitation strategies, propensity to specific infection phenotypes, and pharmacokinetic/pharmacodynamic behavior of antimicrobials and sedatives, arguing against uniform protocols. Heart failure constrains fluid and vasoactive choices; obesity modifies antimicrobial distribution, metabolism, and clearance; COPD adds defective alveolar macrophage function and chronic colonization that predispose to severe pneumonia and ARDS; chronic kidney disease introduces flow–function dissociation, venous congestion, and reduced antimicrobial efficacy; and oncology/HIV contexts complicate recognition and timing because neutropenia, opportunistic pathogens, and atypical biomarker patterns (e.g., low CRP/PCT with elevated IL-10) can mask severity. Intriguingly, severe mental illness has been associated with lower adjusted mortality once sepsis is established, a signal that may reflect pharmacologic or neuroimmune modulation and warrants mechanistic clarification within a precision-sepsis framework.

The current evidence base carries important limitations. Much of it derives from observational, hospital-based cohorts that vary in sepsis definitions, organ-dysfunction scoring, and comorbidity ascertainment, limiting causal inference and generalizability. Neutral results from many immunomodulatory trials likely reflect imprecise patient selection and suboptimal timing within biologically heterogeneous populations rather than a lack of therapeutic potential. Standard bedside scores such as SOFA and qSOFA insufficiently adjust for baseline organ function (notably renal reserve) or for comorbidity-specific physiology, which risks misclassification and delayed intervention. These constraints underscore the need for endotyping approaches that integrate clinical features with immune, endothelial, barrier, and organ-specific biomarkers to guide more accurate selection and timing of therapies.

Clinical implications flow directly from this perspective. Moving toward precision management means aligning interventions with mechanism and phenotype—operationalized in Table 3—by tailoring fluids and vasoactives in patients with limited cardiac or renal reserve; coupling early broad antimicrobial coverage to timely de-escalation, with dose optimization in obesity and chronic kidney disease; and incorporating immune and organ monitoring (e.g., HLA-DR expression or tubular injury biomarkers such as NGAL and KIM-1, where available) to anticipate trajectories. Post-sepsis recovery should likewise be structured with attention not only to cognitive, cardiovascular, respiratory, and renal sequelae, but also to nutritional status, muscle loss, frailty, and emerging osteometabolic vulnerability, all of which may shape long-term functional recovery and survivorship trajectories and are aligned with the patient-specific care pathways outlined in Table 3.

**Table 3:** Comorbidity-tailored management pathways in sepsis—initial resuscitation, antimicrobials, monitoring, organ support, and post-sepsis follow-up.

Comorbidity	Initial resuscitation	Antimicrobials	Monitoring targets	Organ support	Post-sepsis follow-up
T2DM	Avoid hyperglycemia; standard fluids with frequent reassessment.	Weight/volume-of-distribution–aware loading; ensure skin/urinary coverage when applicable.	Blood glucose/ketones; HLA-DR if available.	Thrombosis prevention; usual supportive care.	Nephro-metabolic clinic; diabetic-foot surveillance.
Obesity	Ultrasound-guided, titrated fluids; assess fluid overload early; consider early vasopressor use.	PK/PD optimization: ↑Vd → weight-based loading; consider TDM for narrow-index drugs.	Lactate trend; cumulative fluid balance; respiratory mechanics.	Lung-protective ventilation; DVT prophylaxis (high risk).	Rehabilitation; review/adjust chronic therapies (anticoagulants, antihypertensives, hypoglycemics).
COPD	Cautious fluids; avoid/worsening hypercapnia.	Respiratory/Gram-negative coverage; corticosteroids per COPD-exacerbation guidelines.	Arterial blood gases; SOFA; PaO₂/FiO₂.	Non-invasive ventilation or lung-protective invasive ventilation; consider APRV; prevent barotrauma.	Pulmonology follow-up; exacerbation prevention (vaccination, inhaled therapy, smoking cessation).
CKD	Avoid >20–30 mL/kg without response; use diuresis goals and IVC ultrasound; de-resuscitate when appropriate.	Dose by CrCl; avoid nephrotoxins; use TDM when feasible.	NGAL/KIM-1; urine output; creatinine; cumulative fluid balance.	Decongestive strategy; consider early, selective RRT.	Nephrology follow-up; kidney-protective measures.
HFrEF	Titrated fluids; norepinephrine first-line vasopressor; early diuresis if congested.	Standard broad coverage; check drug–drug interactions.	Echocardiography; lactate; congestion indices (e.g., VExUS/IVC).	Rhythm control as needed; selective inotropes for low-output states.	Cardiac rehabilitation; optimize GDMT (β-blocker, ACEi/ARB/ARNI, MRA, SGLT2i).
Cancer/HIV	Early recognition and prompt source control.	Broad-spectrum plus opportunistic coverage (e.g., antifungal, anti-TB, PJP) tailored to host factors; rapid de-escalation with microbiology.	NLR; IL-10; CD4 count/viral load (HIV); neutrophil count; galactomannan/β-D-glucan as indicated.	Multiorgan supportive care; cautious transfusion strategies.	Oncology/ID follow-up; resume/continue cART when appropriate; monitor for IRIS.

PK/PD, pharmacokinetics/pharmacodynamics; Vd, volume of distribution; TDM, therapeutic drug monitoring; HLA-DR, human leukocyte antigen–DR; DVT, deep-vein thrombosis; SOFA, Sequential Organ Failure Assessment; APRV, airway pressure release ventilation; IVC, inferior vena cava; CrCl, creatinine clearance; NGAL, neutrophil gelatinase-associated lipocalin; KIM-1, kidney injury molecule-1; RRT, renal replacement therapy; VExUS, venous excess ultrasound; GDMT, guideline-directed medical therapy; ACEi, angiotensin-converting-enzyme inhibitor; ARB, angiotensin-receptor blocker; ARNI, angiotensin-receptor–neprilysin inhibitor; SGLT2i, sodium–glucose cotransporter-2 inhibitor; ID, infectious diseases; PJP, Pneumocystis jirovecii pneumonia; cART, combination antiretroviral therapy; IRIS, immune reconstitution inflammatory syndrome. Across all profiles, post-sepsis follow-up should include assessment of nutritional risk, muscle loss/sarcopenia, frailty, and, when appropriate, osteometabolic vulnerability and bone health.

In parallel, health-system priorities must include the prevention of nosocomial sepsis—especially in ICUs and NICUs, where incidence and mortality remain disproportionate—by strengthening surveillance, standardizing clinical/microbiological definitions, and enforcing sustainable infection-prevention bundles. Family- and patient-engagement programs can improve knowledge, preparedness, and escalation behaviors without materially increasing anxiety, even if effects on mortality are modest; the co-design of multimodal education, peer networks, and structured follow-up offers a scalable route to support recovery and continuity of care (122–124) (Table 2).

Future work should emphasize prospective, biomarker-informed endotyping; adaptive trials that test mechanism-matched interventions (e.g., immune-restorative strategies in low HLA-DR phenotypes, antithrombotic/anti-NET approaches in microthrombosis-dominant endotypes, decongestive resuscitation in heart-failure/CKD profiles); dose-finding PK/PD studies in obesity and kidney disease; and longitudinal cohorts quantifying post-sepsis neurocognitive and cardiometabolic outcomes by comorbidity profile, alongside evaluation of structured rehabilitation packages. Clarifying the observed survival signal in severe mental illness and the roles of psychotropic agents and neuroimmune pathways may open novel therapeutic avenues.

Activation of five pathobiological domains across ten chronic comorbidities and their contribution to sepsis vulnerability. Across ten prevalent comorbidities, the activation of five domains—immunity, inflammation, endothelium, barriers, and neuroaxis—to depict shared mechanisms that heighten susceptibility to severe infection and organ failure. “Immunity” denotes impaired innate and adaptive responses that blunt pathogen recognition and clearance; “inflammation” reflects persistent signaling (e.g., NF-κB/JAK–STAT) and immunothrombosis; “endothelium” captures loss of vascular integrity, increased permeability, and procoagulant activation; “barriers” refers to epithelial/mucosal disruption, dysbiosis, and tight-junction failure enabling microbial translocation; and “neuroaxis” indicates neuroendocrine/autonomic dysregulation that fosters immunosuppression and hemodynamic instability. Cells shaded green indicate substantial activation of a given domain within the comorbidity, whereas gray denotes minimal or indirect involvement. By consolidating these signals, the matrix offers a concise, clinically oriented view of systemic vulnerability that supports risk stratification and the design of targeted preventive and therapeutic strategies.

## 11 Conclusion

Sepsis in the setting of multimorbidity is best characterized as a state of systemic vulnerability arising from convergent axes of immune dysregulation, endothelial injury, barrier failure, and neuroendocrine maladaptation. Common comorbidities—type 2 diabetes and obesity, heart failure and cerebrovascular disease, COPD, chronic kidney disease, cancer and HIV, and severe mental illness—not only increase the incidence of sepsis but reshape its clinical trajectory: an early hyperinflammatory phase with immunothrombosis and microvascular injury is followed by immunoparalysis with secondary infections and multiorgan failure. This framework explains the marked bedside heterogeneity, the disproportionate impact of nosocomial sepsis (particularly in ICUs and NICUs), and the substantial burden of neurocognitive and cardiometabolic sequelae among survivors, underscoring the need for integrated strategies spanning prevention, surveillance, and recovery. At the recovery stage, structured follow-up should also consider nutritional risk, sarcopenia, frailty, and bone health as potentially relevant determinants of long-term prognosis after sepsis.

Clinically, progress toward precision sepsis requires aligning interventions with mechanism and phenotype: titrated resuscitation and vasoactive therapy in patients with limited cardiac or renal reserve; pharmacokinetic/pharmacodynamic optimization of antimicrobials in obesity and chronic kidney disease with timely de-escalation; and immune and organ monitoring (e.g., monocyte HLA-DR, NGAL/KIM-1) to anticipate trajectories. At the health-system level, strengthening bundles for prevention of hospital-acquired sepsis and structuring multidisciplinary post-sepsis follow-up are priorities. Important evidence gaps persist due to heterogeneity of definitions and imprecise patient selection and timing. Prospective endotyping and adaptive trials that test mechanism-matched therapies (anti-NET/antithrombotic strategies for microthrombosis-dominant endotypes, decongestive resuscitation in heart-failure/CKD profiles, immune-restorative approaches in low-HLA-DR phenotypes), together with dose-finding PK/PD studies and longitudinal cohorts quantifying neurocognitive and cardiometabolic outcomes, are needed. Framing sepsis through the lens of multimorbidity is essential to reduce mortality and long-term burden and to enable genuinely personalized care.

## Glossary

ACEi: angiotensin-converting enzyme inhibitor
AF: atrial fibrillation
AIDS: acquired immunodeficiency syndrome
AKI: acute kidney injury
AMPK: AMP-activated protein kinase
APACHE II: Acute Physiology and Chronic Health Evaluation II
APRV: airway pressure release ventilation
ARDS: acute respiratory distress syndrome
AUC: area under the curve
BBB: blood–brain barrier
BEX1: brain-expressed X-linked protein 1
BNP: B-type natriuretic peptide
CD4+: cluster of differentiation 4-positive T lymphocyte
CI: confidence interval
CKD: chronic kidney disease
COPD: chronic obstructive pulmonary disease
CRP: C-reactive protein
CrCl: creatinine clearance
CT: computed tomography
CTLA-4: cytotoxic T-lymphocyte–associated protein 4
DAMPs: damage-associated molecular patterns
DVT: deep vein thrombosis
EPHX2: epoxide hydrolase 2
FURIN: furin (proprotein convertase subtilisin/kexin type 3)
GDMT: guideline-directed medical therapy
GNMT: glycine N-methyltransferase
HF: heart failure
HFrEF: heart failure with reduced ejection fraction
HIV: human immunodeficiency virus
HLA-DR: human leukocyte antigen–DR
ICU: intensive care unit
ICUs: intensive care units
ID: infectious diseases
IL-1β: interleukin-1β
IL-6: interleukin-6
IL-10: interleukin-10
IRIS: immune reconstitution inflammatory syndrome
IVC: inferior vena cava
JAK–STAT: Janus kinase–signal transducer and activator of transcription
JNK: c-Jun N-terminal kinase
KIM-1: kidney injury molecule-1
LPS: lipopolysaccharide
MRSA: methicillin-resistant *Staphylococcus aureus*
MRA: mineralocorticoid receptor antagonist
NETosis: neutrophil extracellular trap–mediated cell death
NETs: neutrophil extracellular traps
NGAL: neutrophil gelatinase-associated lipocalin
NICU: neonatal intensive care unit
NLR: neutrophil-to-lymphocyte ratio
NOD: nucleotide-binding oligomerization domain
NT-proBNP: N-terminal pro–B-type natriuretic peptide
OR: odds ratio
PAMPs: pathogen-associated molecular patterns
PCT: procalcitonin
PD-1: programmed cell death protein 1
PJP: pneumocystis jirovecii pneumonia
PK/PD: pharmacokinetics/pharmacodynamics
RAAS: renin–angiotensin–aldosterone system
RNASE2: ribonuclease A family member 2
RRT: renal replacement therapy
SGLT2i: sodium–glucose cotransporter 2 inhibitor
SIRT1: sirtuin 1
SMI: severe mental illness
SOFA: Sequential Organ Failure Assessment
T2DM: type 2 diabetes mellitus
TB: tuberculosis
TDM: therapeutic drug monitoring
TLR: Toll-like receptor
TNF-*α*: tumor necrosis factor-α
Th1: T helper 1
Th17: T helper 17
VExUS: venous excess ultrasound
Vd: volume of distribution
cART: combination antiretroviral therapy
qSOFA: quick Sequential Organ Failure Assessment
